# Prognostic value of androgen receptor expression in ER-positive/HER2-negative breast cancer: evidence from a contemporary Chinese cohort

**DOI:** 10.3389/fonc.2026.1824532

**Published:** 2026-05-11

**Authors:** Cheng Tian, Lizheng Xiang, Shengchun Liu, Haochen Yu

**Affiliations:** Department of Breast and Thyroid Surgery, Chongqing Key Laboratory of Molecular Oncology and Epigenetics, The First Affiliated Hospital of Chongqing Medical University, Chongqing, China

**Keywords:** androgen receptor, breast cancer, estrogen receptor, neoadjuvant treatment, progesterone receptor

## Abstract

**Background:**

The prognostic significance of androgen receptor (AR) expression in ER+/HER2− luminal breast cancer remains controversial, particularly in the context of neoadjuvant therapy. Most available evidence is derived from Western populations, and data from contemporary Asian cohorts are limited. This study aimed to evaluate the association between AR expression and survival outcomes in a real-world Chinese cohort.

**Methods:**

We retrospectively analyzed 422 patients with primary ER+/HER2− breast cancer treated at the First Affiliated Hospital of Chongqing Medical University between December 2020 and November 2021. AR expression was assessed by immunohistochemistry, and patients were stratified using a predefined cutoff. Disease-free survival (DFS) and overall survival (OS) were estimated by the Kaplan–Meier method and compared with the log-rank test. Cox proportional hazards models were used to identify factors associated with DFS. Exploratory subgroup analyses were performed among patients receiving neoadjuvant chemotherapy, particularly those without pathological complete response (pCR).

**Results:**

Higher AR expression was significantly associated with prolonged DFS, whereas no significant association with OS was observed during the current follow-up period. In multivariable analysis, baseline tumor T stage, progesterone receptor expression, and AR expression were independently associated with DFS. Among patients treated with neoadjuvant chemotherapy who did not achieve pCR, AR expression was also associated with DFS. Sensitivity analyses treating AR as a continuous variable showed directionally consistent but non-significant results.

**Conclusions:**

AR expression may have prognostic value in ER+/HER2− breast cancer, particularly for DFS and in selected patients with residual disease after neoadjuvant chemotherapy. Further prospective studies with longer follow-up are warranted.

## 1 Introduction

Breast cancer is the most common malignancy and the second leading cause of cancer-related death among women worldwide (1). Based on immunohistochemical indicators, breast cancer is classified into four molecular subtypes: Luminal A, Luminal B, HER2-positive, and basal-like (2). Among these, luminal-type breast cancer, defined by ER positivity, represents approximately 60–70% of all cases (3, 4). According to the international consensus of experts from St. Gallen, Luminal-type breast cancer can be further classified into Luminal A and Luminal B subtypes based on HER2 status, progesterone receptor (PR) expression, and the Ki-67 proliferation index. As the most prevalent subtype of breast cancer, luminal disease has attracted substantial clinical attention with respect to its treatment and prognosis, because its high incidence is accompanied by a persistent risk of recurrence and death: luminal A and luminal B tumors show a continuous pattern of recurrence over time, ER-positive disease remains at risk of distant recurrence even 5–20 years after completion of 5 years of endocrine therapy, and luminal B tumors are associated with significantly worse breast cancer-specific survival than luminal A tumors (5–8).

The androgen Receptor (AR), alongside the ER and PR, is a key member of the steroid hormone receptor superfamily, playing a pivotal role in breast cancer tumorigenesis and progression (9). The mechanism of action and clinical application of AR in breast cancer have been reported. In ER-positive breast cancer, there is a complex interplay and regulatory crosstalk between AR and ER; AR can counteract the ER-mediated transcription of proliferation-related genes by competing with DNA regulatory elements and co-activators, thereby inhibiting estrogen-driven tumor growth (10). However, under certain changes in the hormonal environment or the stress of endocrine therapy, AR signaling can redirect the transcriptional program, thereby promoting the survival and progression of tumor cells (11, 12). The dual functions of AR reflect the complexity of its interaction with ER in luminal-type breast cancer. Previous retrospective studies have found that the interaction between ER and AR, to some extent, may inhibit the progression of breast cancer (13, 14). Despite these findings, the clinical role of AR in Luminal-type breast cancer (LBC) remains controversial. Previous studies have found that, in LBC patients, high AR expression is negatively correlated with the efficacy of neoadjuvant therapy (15). Previous clinical studies have shown that high AR expression may be associated with inferior neoadjuvant efficacy in luminal breast cancer. In the GeparTrio cohort, AR-positive tumors achieved a significantly lower pCR rate than AR-negative tumors (12.8% vs. 25.4%, P<0.0001), while another study of ER-positive breast cancer further showed that AR-high tumors consistently exhibited lower pCR rates across ten neoadjuvant chemotherapy cohorts (5, 16). Large cohort studies and gene expression analyses have also found that AR expression is significantly associated with lower proliferative characteristics and longer long-term survival in LBC (17). Furthermore, the majority of existing data are derived from Western populations. Given that genetic backgrounds, hormonal environments, and treatment responses can vary across ethnicities, data from Asian populations remain scarce. Therefore, elucidating the prognostic role of AR in Chinese patients is essential to validate Western findings and refine risk stratification strategies for this specific demographic.

This single-center retrospective study analyzed patients with Luminal-type breast cancer at the First Affiliated Hospital of Chongqing Medical University. It aimed to explore the impact of AR expression on the long-term survival outcomes of Luminal-type, HER2-negative breast cancer patients.

## 2 Materials and methods

### 2.1 Patients

This study included a total of 422 patients with primary Luminal-type breast cancer who were treated at the Breast and Thyroid Surgery Department of the First Affiliated Hospital of Chongqing Medical University from December 2020 to November 2021. The specific inclusion criteria are as follows: (1) patients have an Eastern Cooperative Oncology Group (ECOG) performance status score of 0 to 1; (2) patients have been diagnosed with invasive breast cancer through pathological examination of biopsy samples; (3) immunohistochemistry (IHC) results consistent with the Luminal-type molecular subtype criteria: estrogen receptor-positive and human epidermal growth factor receptor 2-negative.

**Figure 1:**
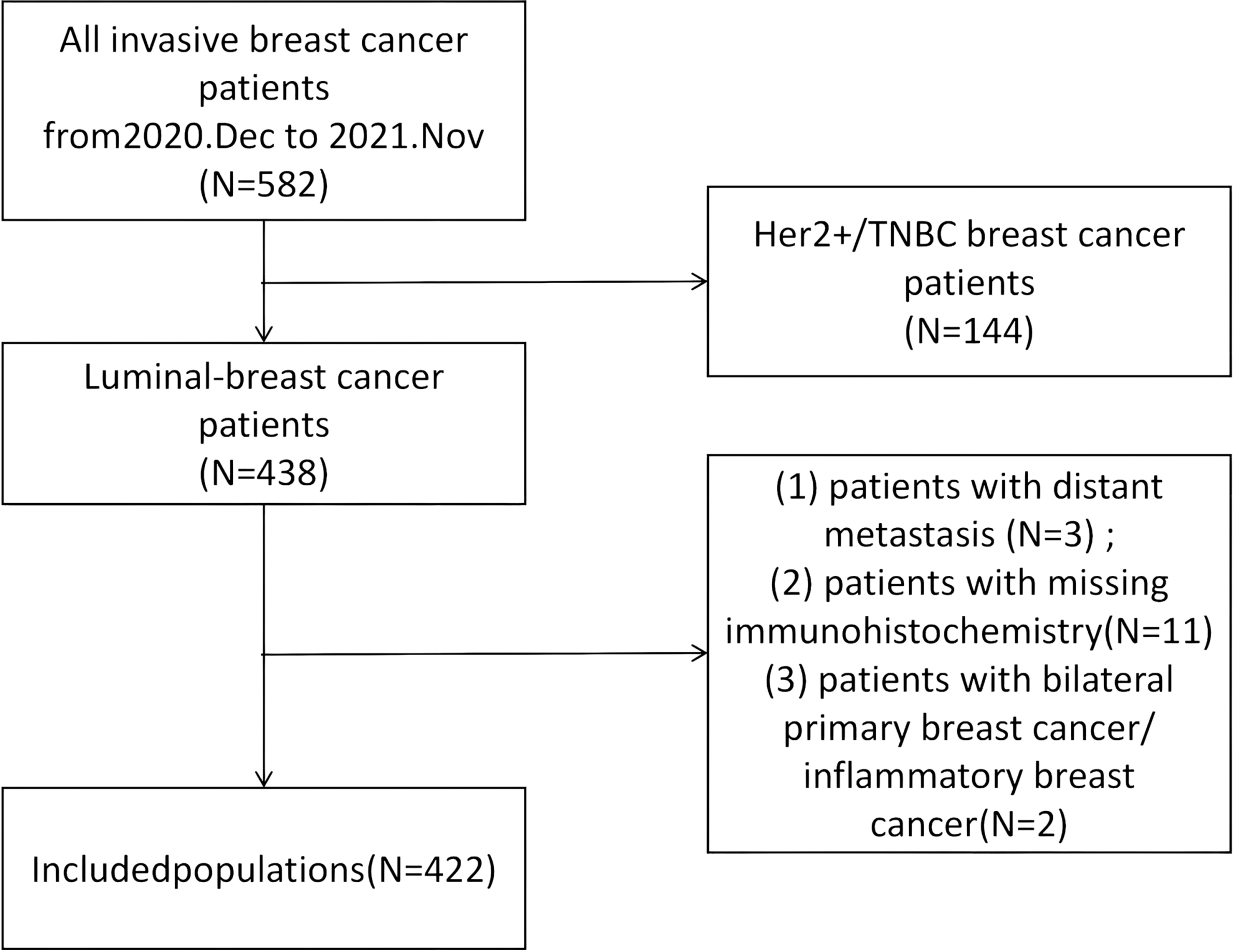
Flowchart of patient recruitment and selection. A total of 582 patients with invasive breast cancer diagnosed between December 2020 and November 2021 were initially screened. Patients with HER2-positive or triple-negative breast cancer (TNBC) subtypes were excluded (N = 144). From the remaining 438 patients with Luminal-type breast cancer, those with distant metastasis (N = 3), missing immunohistochemistry data (N = 11), or bilateral/inflammatory breast cancer (N = 2) were further excluded. Ultimately, 422 patients were included in the final analysis.

The exclusion criteria are as follows: (1) patients with distant metastasis at the initial diagnosis; (2) patients with missing immunohistochemistry data in the pathology report that cannot be re-obtained; (3) patients who have previously undergone surgical treatment before study enrollment or have incomplete postoperative follow-up data; (4) patients with bilateral primary breast cancer and inflammatory breast cancer (**Figure 1**).

### 2.2 Specimen preparation and staining

Breast tissue specimens were fixed in 10% neutral buffered formalin, dehydrated through a graded alcohol series, and embedded in paraffin according to standardized tissue-processing procedures. Hematoxylin and eosin(H&E)-stained sections were reviewed independently by two associate chief pathologists, each with more than 10 years of experience in breast pathology. Histopathological diagnosis was based on the latest World Health Organization (WHO) classification of breast tumors and the Nottingham histological grading system. Both pathologists were blinded to the patients’ clinical and imaging information during evaluation.

Immunohistochemical staining for ER, PR, HER2, AR, and Ki-67 was performed on formalin-fixed, paraffin-embedded tissue sections. The primary antibodies were as follows: ER (clone SP1, Roche, ready-to-use), PR (clone 1E2, Roche, ready-to-use), HER2 (clone 4B5, Roche, ready-to-use), AR (clone EP120, Celnotive, ready-to-use), and Ki-67 (clone 30-9, Roche, ready-to-use). Antigen retrieval was performed using phosphate-buffered saline (PBS)-based buffer (pH 7.4–7.6) at room temperature, with marker-specific retrieval conditions according to routine laboratory protocols. ER, PR, and HER2 staining were performed on the UltraView platform, whereas AR and Ki-67 staining were performed manually.

According to the 2020 ASCO/CAP guidelines (18), ER and PR positivity were defined as positive nuclear staining in ≥1% of tumor cell nuclei, whereas staining in <1% of tumor cell nuclei was considered negative. HER2 immunohistochemical expression was scored according to the 2018 ASCO/CAP guidelines as 0, 1+, 2+, or 3+; a score of 3+ was considered positive, 0–1+ negative, and 2+ equivocal, requiring further evaluation by fluorescence *in situ* hybridization (FISH). AR expression was assessed as the percentage of positively stained tumor cell nuclei. AR positivity was defined as nuclear staining in ≥1% of tumor cells. Ki-67 was evaluated as the percentage of tumor cells showing positive nuclear staining.

FISH testing was performed for all HER2 IHC 2+ cases and for selected IHC 1+ or 3+ cases with clinical suspicion of HER2 positivity, using a HER2 dual-probe kit (Guangzhou ABP Biotech Co., Ltd., Guangzhou, China). HER2 gene amplification was interpreted according to the kit instructions and contemporary ASCO/CAP criteria. HER2 positivity was defined as a HER2/CEP17 ratio ≥2.0 with an average HER2 copy number ≥4.0 signals/cell, or a HER2/CEP17 ratio <2.0 with an average HER2 copy number ≥6.0 signals/cell.

In patients with residual invasive carcinoma after neoadjuvant therapy, immunohistochemical reassessment of ER, PR, HER2, and Ki-67 was performed on residual tumor tissue as part of routine post-neoadjuvant pathological evaluation. This practice is consistent with guideline-based pathology recommendations in China, as biomarker expression may change after systemic therapy and updated post-treatment receptor status may affect subsequent adjuvant treatment decisions, particularly endocrine therapy and anti-HER2 therapy planning. In contrast, AR was not routinely reassessed in residual tumor tissue, because it is not currently included among the standard biomarkers required for routine post-neoadjuvant pathological evaluation in current clinical practice.

Representative IHC images showing positive and negative staining patterns, as well as a representative FISH image from a HER2 IHC 2+ case, are provided in the **Supplementary Figure 1**.

### 2.3 Treatment regimens and outcomes

The neoadjuvant treatment plan was developed based on the guidelines for breast cancer diagnosis and treatment of the Chinese Society of Clinical Oncology (CSCO). According to CSCO recommendations, preoperative systemic therapy may be considered for patients with a primary tumor >5 cm, axillary node-positive disease, HER2-positive or triple-negative breast cancer, or when downstaging is needed to facilitate breast-conserving surgery.

The preferred chemotherapy regimen includes the following drugs: (1) T: docetaxel 75 mg/m² intravenous infusion once every 3 weeks or albumin-bound paclitaxel 200–260 mg/m² intravenous infusion once every 3 weeks; (2) A: pirarubicin 50 mg/m² intravenous infusion once every 3 weeks; (3) C: cyclophosphamide 500 mg/m² intravenous infusion once every 3 weeks. After every two treatment cycles, an efficacy assessment is conducted for the patients. This assessment mainly involves breast ultrasound examination to dynamically monitor changes in the lesions. All patients undergo surgical treatment after completing the predetermined neoadjuvant therapy regimen, and the postoperative pathological response is evaluated using the Miller-Payne scoring system. Pathological complete response (pCR) is defined as the absence of any residual invasive cancer in the breast and axillary lymph nodes; however, if there is still residual ductal carcinoma *in situ* (DCIS) within the breast, it is still regarded as meeting the criteria for pathological complete response (19).

Postoperative endocrine therapy was administered according to guideline-based routine practice and individualized according to menopausal status and recurrence risk. For postmenopausal patients with hormone receptor–positive disease, aromatase inhibitor–based endocrine therapy was generally preferred as the main adjuvant endocrine strategy. For premenopausal patients, tamoxifen-based therapy was generally used in lower-risk settings, whereas ovarian function suppression (OFS) combined with tamoxifen or with an aromatase inhibitor was considered for higher-risk patients, particularly those with greater tumor burden or other adverse clinicopathological features.

### 2.4 Statistical analysis

In this study, comparisons of clinical and pathological characteristics among different groups were conducted. For continuous variables, independent sample t-tests were used; for categorical variables, Pearson’s χ² test or Fisher’s exact test was employed; and for ordinal variables, the Mann-Whitney U test was applied. The analysis of factors influencing pathological reactions employed the binary logistic regression model to calculate the corresponding odds ratio (OR). Meanwhile, the analysis of predictors of long-term survival utilized the Cox proportional hazards regression model to calculate the hazard ratio (HR). In the binary logistic regression and Cox regression analyses, all continuous variables were appropriately grouped according to clinical guidelines and experience to better interpret the results. For analyses using dichotomized receptor expression, tumors with ER, PR, or AR nuclear staining in >30% of tumor cells were classified as high-expression, whereas those with staining in ≤30% of tumor cells were classified as low-expression. These analytical categories were used for survival stratification and should be distinguished from the pathological definition of receptor positivity, which was based on a ≥1% cutoff. Disease-free survival (DFS) was defined as the interval from the date of breast cancer diagnosis to the first occurrence of recurrence, metastasis, or death, or was censored at the last follow-up. Overall survival (OS) was defined as the time from the date of diagnosis to death due to any cause or was censored at the last follow-up. Both DFS and OS analyses were conducted using the Kaplan-Meier method to estimate survival curves, and log-rank tests were employed to compare survival differences among groups. All statistical tests were two-sided, and a p-value<0.05 was considered statistically significant. All statistical analyses were completed using the R software package.

## 3 Results

### 3.1 Patient characteristics

This study included a total of 422 female breast cancer patients who were initially diagnosed with luminal-type (HER2-negative). The average age of the patients was 51.3 years. Regarding body mass index(BMI) distribution, 60.2% of the patients (N = 254) had a BMI of 24 kg/m² or less, while 39.8% (N = 168) had a BMI greater than 24 kg/m². The distribution of menopausal status was relatively balanced, with 51.9% of the patients being postmenopausal. Concerning tumor pathological characteristics, invasive ductal carcinoma was the predominant histological type, accounting for 91.9% of the patients. The histological grade was predominantly G2, accounting for 84.8% (N = 358), while G1 and G3 accounted for 2.6% and 12.6%, respectively. The distribution of molecular subtypes showed that the proportions of Luminal A and Luminal B patients were 48.6% (N = 205) and 51.4% (N = 217), respectively. With respect to clinical staging, tumors at stage cT2 were the most common (53.3%), and 87.9% of the patients had no clinical lymph node metastasis (cN0).

The baseline characteristics of the patients were stratified and compared based on whether they received neoadjuvant therapy (**Table 1**). There were significant statistical differences found in multiple clinical and pathological indicators between the two groups. The average age of patients in the neoadjuvant therapy group (47.7 years) was significantly lower than that of the non-neoadjuvant therapy group (51.9 years, P = 0.007). Regarding molecular characteristics, the proportion of Luminal B patients in the neoadjuvant therapy group was significantly higher (84.6%vs.45.4%, P<0.001).The distribution of PR categories differed significantly between the neoadjuvant and non-neoadjuvant groups (P = 0.006), with a lower proportion of tumors showing PR >30% in the neoadjuvant group (52.3% vs. 67.5%). The average Ki-67 index was also significantly higher than that of the non-neoadjuvant therapy group (32.7%vs.21.4%, P<0.001). Furthermore, there were significant differences in the distribution of clinical T stages (cT stages, P<0.001), lymph node stages (cN stages, P<0.001), and axillary lymph node (ALN) metastasis (P<0.001) between the two groups. Other indicators, including BMI, menopausal status, histological type, and HER2 status, did not show statistically significant differences between the two groups (P>0.05).

**Table 1:** Baseline characteristics of the luminal BC study population.

Characteristic	Total	Neoadjuvant	Non-Neoadjuvant	P value
Age (years)				0.007
Mean(SD)	51.3(11.7)	47.7(11.6)	51.9(11.6)	
BMI (kg/m²)				0.390
<=24	254(60.2%)	36(55.4%)	218(61. 1%)	
>24	168(39.8%)	29(44.6%)	139(38.9%)	
Menopausal Status				0.202
Postmenopausal	219(51.9%)	29(44.6%)	190(53.2%)	
Premenopausal	203(48.1%)	36(55.4%)	167(46.8%)	
Histology Type				0.482
Invasive ductal carcinoma	388(91.9%)	63(96.9%)	325(91.0%)	
Invasive lobular carcinoma	17(4.0%)	2(3.1%)	15(4.2%)	
Metaplastic carcinoma	1(0.2%)	0(0.0%)	1(0.3%)	
Mucinous carcinoma	11(2.6%)	0(0.0%)	11(3.1%)	
Papillary carcinoma	5(1.2%)	0(0.0%)	5(1.4%)	
Histological Grade				0.262
G1	11(2.6%)	1(1.5%)	10(2.8%)	
G2	358(84.8%)	52(80.0%)	306(85.7%)	
G3	53(12.6%)	12(18.5%)	41(11.5%)	
Molecular Subtype				<0.001
LuminalA	205(48.6%)	10(15.4%)	195(54.6%)	
LuminalB	217(51.4%)	55(84.6%)	162(45.4%)	
cT Stage				<0.001
T1	182(43.1%)	9(13.8%)	173(48.5%)	
T2	225(53.3%)	49(75.4%)	176(49.3%)	
T3	10(2.4%)	4(6.2%)	6(1.7%)	
T4	5(1.2%)	3(4.6%)	2(0.6%)	
cN Stage				<0.001
N0	371(87.9%)	29(44.6%)	342(95.8%)	
N1	47(11.1%)	33(50.8%)	14(3.9%)	
N2	2(0.5%)	1(1.5%)	1(0. 3%)	
N3	2(0.5%)	2( 3.1%)	0(0.0%)	
ALN Metastasis				<0.001
No	11(2.6%)	2(3.1%)	9(2.5%)	
Unknown	361(85.5%)	26(40.0%)	335(93.8%)	
Yes	50(11.8%)	37(56.9%)	13(3.6%)	
ER Expression(%)				0.214
<1%	9(2.1%)	2(3.1%)	7(2.0%)	
1-10%	12(2.8%)	4(6.2%)	8(2.2%)	
11-30%	7(1.7%)	2(3.1%)	5(1.4%)	
>30%	394(93.4%)	57(87.7%)	337(94.4%)	
PR Expression(%)				0.006
<1%	51(12.1%)	12(18.5%)	39(10.9%)	
1-10%	43(10.2%)	13(20.0%)	30(8.4%)	
11-30%	53(12.6%)	6(9.2%)	47(13.2%)	
>30%	275(65.2%)	34(52.3%)	241(67.5%)	
HER2 Status				0.129
0	130(30.8%)	18(27.7%)	112(31.4%)	
1+	184(43.6%)	23(35.4%)	161(45.1%)	
2+Fish-	107(25.4%)	24(36.9%)	83(23.2%)	
2+Fish+	1(0.2%)	0(0.0%)	1(0.3%)	
Ki-67 Index(%)				<0.001
Mean (SD)	23.2(15.6)	32.7(18.6)	21.4(14.3)	
AR Expression(%)				0.942
<1%	175(41.5%)	26(40.0%)	149(41.7%)	
1-10%	67(15.9%)	12(18.5%)	55(15.4%)	
11-30%	61(14.5%)	9(13.8%)	52(14.6%)	
>30%	119(28.2%)	18(27.7%)	101(28.3%)	
AR/ER Ratio				0.136
<=1	401(97.1%)	63(100.0%)	338(96.6%)	
>1	12(2.9%)	0(0.0%)	12(3. 4%)	
AR/PR Ratio				0.855
<=1	319(86.0%)	46(86.8%)	273(85.8%)	
>1	52(14.0%)	7(13.2%)	45(14.2%)	

BC, Breast Cancer; ALN, Axillary Lymph Node; BMI, Body Mass Index.

Of the total patients, 65 received neoadjuvant therapy (**Table 2**). The TEC regimen (docetaxel + epirubicin + cyclophosphamide) was the main chemotherapy protocol, used in 95.4% (N = 62) of the patients. The remaining few patients received the EC-T or TP regimens. Regarding the assessment of pathological responses after treatment, the Miller-Payne classification revealed that G3 was the most common response grade (40.0%, N = 26), followed by G2 (24.6%, N = 16) and G5 (16.9%, N = 11). A total of 13.8% (N = 9) of the patients achieved pCR. The postoperative pathological staging indicated that the majority of patients (80.0%, N = 52) were in the ypT1–2 stage, while 16.9% (N = 11) were in the ypT0 stage. In terms of lymph node status, 41.5% (N = 27) of the patients achieved ypN0 (no lymph node metastasis), while 58.5% (N = 38) remained ypN+. The analysis of biological markers for residual tumor tissues revealed that the estrogen receptor (ER) was mainly highly expressed, with 67.7% (N = 44) of the patients having ER levels≥30%; the proportion of patients with progesterone receptor (PR) expression≥30% was 30.8% (N = 20). Furthermore, 58.5% (N = 38) of the patients had their residual tumor Ki67 index maintained at ≤ 20%. Further analysis of the dynamic changes of Ki67 (ΔKi67) revealed that 49.2% (N = 32) of the patients showed a slight decrease or no change. Only 9.2% (N = 6) showed a significant decrease, and another 7.7% (N = 5) experienced an increase in the Ki-67 index after treatment.

**Table 2:** Characteristics of neoadjuvant therapy patients.

Clinicopathologic Feature	No.of Patients(%)
Neoadjuvant therapy	
EC-T	2(3.1%)
TEC	62(95.4%)
TP	1(1.5%)
Miller-Payne stage	
G1	3(4.6%)
G2	16(24.6%)
G3	26(40.0%)
G4	9(13.8%)
G5	11(16.9%)
pCR	
No	56(86.2%)
Yes	9(13.8%)
ypT stage	
ypT0	11(16.9%)
ypT1-2	52(80.0%)
ypT3-4	2(3.1%)
ypN stage	
ypN0	27(41.5%)
ypN+	38(58.5%)
ER of residual tumor	
1-10%	1(1.5%)
11-30%	4(6.2%)
≥ 30%	44(67.7%)
pCR/unknown	16(24.6%)
PR of residual tumor	
1-10%	11(16.9%)
11-30%	6(9.2%)
≥30%	20(30.8%)
pCR/unknown	28(43.1%)
Ki67 of residual tumor	
Ki67≤20%	38(58.5%)
Ki67≤20%	14(21.5%)
pCR/unknown	13(20.0%)
△Ki67	
Large decrease	6(9.2%)
Medium decrease	9(13.8%)
Slight decrease/no change	32(49.2%)
Increase	5(7.7%)
pCR/Unknown	13(20.0%)

### 3.2 Risk factors for disease-free survival in the overall luminal breast cancer population

**Table 3** presents the results of univariate and multivariate Cox proportional hazards regression analyses for disease-free survival (DFS). Univariate analysis revealed that tumor size (P<0.001), baseline T stage (P = 0.004), baseline N stage (P = 0.008), as well as the expression levels of ER, PR, Ki67, AR, and whether neoadjuvant therapy was received was significantly associated with DFS (all P<0.05). After incorporating these factors into a multivariate analysis, the results indicated that baseline T stage, PR, and AR were independent prognostic factors independently associated with DFS. Specifically, patients with T3–4 stage had a significantly higher risk of recurrence compared to those with T0–2 stage (HR = 5.19, 95% confidence interval(CI): 1.62–16.7, P = 0.016). In terms of hormone receptors, low expression of PR (≤10%) was independently associated with poorer DFS (HR = 3.75, 95% CI: 1.25–11.3, P = 0.043). Furthermore, an AR expression level of ≤30% was also identified as an independent risk factor for DFS (HR = 6.55, 95% CI: 0.86–49.9, P = 0.017), although the wide confidence interval suggests caution in interpreting this result.

**Table 3:** Univariate and multivariate cox regression analysis for DFS.

Characteristic	Univariate Analysis			Multivariate Analysis		
	HR	95%CI	p-value	HR	95%CI	p-value
Age			0.487			0.848
<=40						
>40	0.69	(0.25,1.89)		1.11	(0.37,3.38)	
BMI			0.104			
<=24						
>24	2.04	(0.86,4.84)				
Menopause			0.624			
Premenopausal			
Postmenopausal	1.24	(0.52,2.94)	
Tumor size			<0.001			
<=20 mm			
21-50 mm	5.24	(1.52,18.1)	
>50 mm	18.8	(3.79,93.1)	
Multifocality			0.859			
Unifocal			
Multifocal	0.92	(0.36,2.37)	
Baseline T stage			0.004			0.016
T0-2						
T3-4	7.13	(2.40,21.2)		5.19	(1.62,16.7)	
Baseline N stage			0.008			0.551
N0						
N+	3.85	(1.55,9.54)		1.49	(0.40,5.49)	
Grade			0.068			
G1-2			
G3	2.60	(1.01,6.70)	
Molecular subtype			0.056			0.689
LuminalA						
LuminalB	2.41	(0.93,6.20)		0.78	(0.23,2.63)	
ER			0.022			
>30%			
11-30%	7.90	(1.81,34.4)	
<=10%	3.88	(1.13,13.3)	
PR			0.001			0.043
>30%						
11-30%	3.49	(0.99,12.4)		2.89	(0.79,10.6)	
<=10%	5.73	(2.12,15.5)		3.75	(1.25,11.3)	
Ki67			0.047			0.302
<=30%						
>30%	2.56	(1.06,6.18)		1.74	(0.61,4.94)	
AR			0.005			0.017
>30%						
<=30%	8.11	(1.09,60.4)		6.55	(0.86,49.9)	
AR/ER			0.679			
<=1			
>1	1.57	(0.21,11.8)	
AR/PR			0.583			
<=1			
>1	1.38	(0.45,4.24)	
Neoadjuvant therapy			0.009			0.340
No						
Yes	3.56	(1.48,8.59)		1.910.52,7.04	(0.52,7.04)	

HR, Hazard Ratio; CI, Confidence Interval.

### 3.3 Sensitivity analysis

When AR expression was analyzed as a continuous variable, no significant association with DFS was observed per 1% increase (HR = 0.98, 95% CI: 0.97–1.00; P = 0.84). When scaled per 10% increase, higher AR expression showed a non-significant trend toward improved DFS (HR = 0.86, 95% CI: 0.70–1.04; P = 0.084).

### 3.4 Prognosis-related risk factors in the luminal (HER2-negative) breast cancer population who accepted neoadjuvant treatment

As shown in **Table 4**, we conducted a prognostic stratification analysis based on pCR status among patients who received neoadjuvant chemotherapy. Patients achieving pCR demonstrated excellent disease-free survival (DFS), with no recurrence events observed during follow-up, whereas the DFS rate in the non-pCR group was 85.71%. Overall survival (OS) rates were 88.89% in the pCR group and 94.64% in the non-pCR group.

**Table 4:** Prognostic analysis of patients in the pCR and non-pCR groups.

Group	DFS	OS
pCR	100.00%	88.89%
non-pCR	85.71%	94.64%

To explore potential factors associated with recurrence among non-pCR patients, a univariate Cox regression analysis was performed in this subgroup (**Table 5**). Most clinicopathological variables, including age, BMI, clinical T and N stage, histological grade, ER, PR, Ki67, and Miller–Payne grading, were not significantly associated with DFS (P>0.05). Baseline androgen receptor (AR) expression showed an association with DFS in non-pCR patients (P = 0.026). Given the limited sample size and number of events, these results should be interpreted descriptively.

**Table 5:** Univariate analysis of prognostic factors for DFS (non-pCR).

Characteristic	HR	95%CI	p-value
Age			0.433
<=40			
>40	0.57	(0.14,2.29)	
BMI			0.576
<=24			
>24	1.49	(0.37,5.95)	
Menopause			0.240
Premenopausal			
Postmenopausal	2.32	(0.55,9.69)	
Baseline T			0.202
T1-2			
T3-4	3.20	(0.64,15.9)	
Baseline N			0.685
N0			
N+	0.75	(0.19,3.00)	
Baseline Grade			0.627
G1-2			
G3	1.51	(0.30,7.48)	
Subtype			0.691
LuminalA			
LuminalB	1.50	(0.18,12.2)	
Baseline ER			0.345
>10%			
<=10%	3.21	(0.39,26.2)	
Baseline PR			0.080
>10%			
<=10%	3.50	(0.84,14.7)	
Baseline Ki67			0.424
<=30%			
>30%	1.77	(0.44,7.07)	
Baseline AR			0.026
>30%			
<=30%	3	(0.34,22.4)	
Miller-Payne Grade			0.564
MP1-3			
MP4-5	0.56	(0.07,4.58)	
ypN Stage			0.659
ypN0			
ypN+	1.42	(0.29,7.04)	
Residual AR(30%cutoff)			0.283
>30%			
≤30%	2.76	(0.34,22.4)	
Residual ER(30%cutoff)			0.404
>30%			
≤30%	2.07	(0.42,10.3)	
Residual PR(30%cutoff)			0.076
>30%			
≤30%	4.88	(0.60,39.7)	
Residual Ki67			0.487
≤20%			
>20%	1.68	(0.40,7.05)	
Ki67 Change			0.186
Decrease			
No Decrease	0.00	(0.00,Inf)	

HR, Hazard Ratio; CI, Cofidence Interval.

### 3.5 Survival analysis

**Figure 2:**
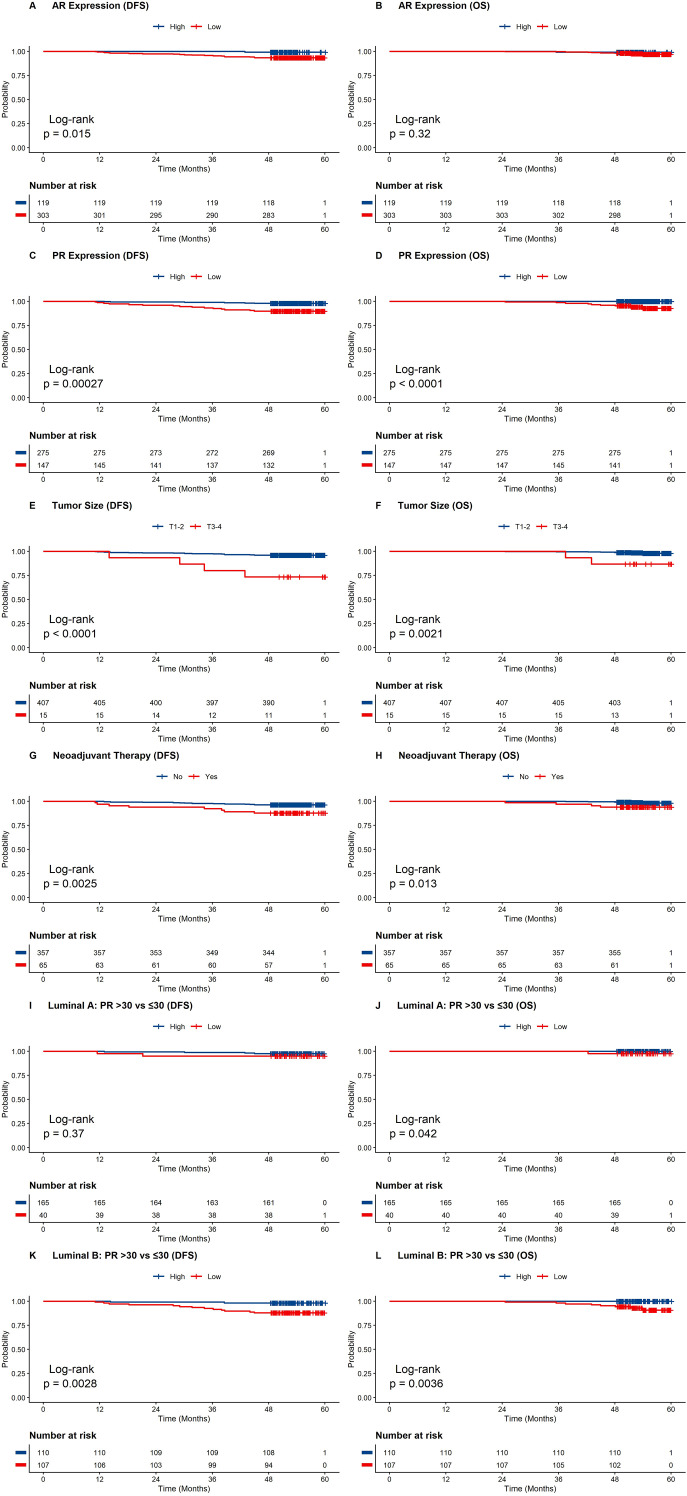
Kaplan–Meier analyses of disease-free survival (DFS) and overall survival (OS) in patients with luminal (HER2-negative) breast cancer. **(A, B)** DFS and OS according to AR expression. **(C, D)** DFS and OS according to PR expression (>30% vs. ≤30%) in the overall cohort. **(E, F)** DFS and OS according to tumor size. **(G, H)** DFS and OS according to receipt of neoadjuvant therapy. **(I, J)** Exploratory subtype-stratified DFS and OS analyses according to PR expression (>30% vs. ≤30%) in Luminal A tumors. **(K, L)** Exploratory subtype-stratified DFS and OS analyses according to PR expression (>30% vs. ≤30%) in Luminal B tumors.

The median follow-up time for the patients was 1620 days. **Figure 2** presents the Kaplan–Meier survival curves for DFS and OS stratified by major clinicopathological variables and biomarker expression. In the overall cohort, patients with high AR expression had significantly better DFS than those with low AR expression (**Figure 2A**, P = 0.015), whereas no statistically significant difference in OS was observed (**Figure 2B**, P = 0.32). PR expression was also significantly associated with survival in the overall cohort. Patients with PR-high tumors showed better DFS (**Figure 2C**, P = 0.00027) and OS (**Figure 2D**, log-rank P < 0.0001) than those with PR-low tumors. Patients with smaller tumors (T1–2) had significantly better DFS and OS than those with larger tumors (T3–4) (**Figure 2E**, P < 0.0001; **Figure 2F**, P = 0.0021). Similarly, patients who did not receive neoadjuvant therapy showed significantly better DFS and OS than those who received neoadjuvant therapy (**Figure 2G**, P = 0.0025; **Figure 2H**, P = 0.013).

To further explore whether the prognostic relevance of AR and PR differed by luminal subtype, additional exploratory Kaplan–Meier analyses were performed separately in Luminal A and Luminal B tumors (**Supplementary Figure 2**). In the Luminal A subgroup, AR expression was not significantly associated with either DFS or OS (**Supplementary Figure 2A**, P = 0.46; **Supplementary Figure 2B**, P = 0.51). PR expression in Luminal A tumors was not significantly associated with DFS (**Supplementary Figure 2C**, P = 0.37), whereas the OS curves showed limited separation (**Supplementary Figure 2D**, P = 0.042), which should be interpreted cautiously given the very small number of death events. In the Luminal B subgroup, high AR expression was associated with more favorable DFS (**Supplementary Figure 2E**, P = 0.018), but no significant difference in OS was observed (**Supplementary Figure 2F**, P = 0.46). In contrast, PR-high tumors in the Luminal B subgroup showed significantly better DFS and OS than PR-low tumors (**Supplementary Figure 2G**, P = 0.0028; **Supplementary Figure 2H**, P = 0.0036).

## 4 Discussion

Unlike triple-negative breast cancer, which is characterized by a recurrence peak within the first three years, LBC exhibits a lower initial risk but follows a bimodal recurrence pattern over a 20-year period, with two distinct peaks observed at 2–3 years and 8–9 years post-diagnosis (6, 20). As the most common molecular subtype of breast cancer, LBC has long been a concern for clinicians regarding patient prognosis, due to its long-term recurrence risk and heterogeneous prognosis.

AR, together with ER and PR, belongs to the steroid receptor family. Historically, AR has been largely overshadowed by the extensive clinical focus on ER and PR. However, accumulating evidence is now unveiling its critical involvement in the initiation and progression of breast cancer. Previous studies have reported the potential clinical value of AR in triple-negative breast cancer (21). However, the role of AR in LBC cannot be simply dichotomized into a protective factor or a risk factor. Instead, its clinical significance is highly context-dependent, varying according to the specific therapeutic regimens and its expression. Although the prognostic role of AR expression in ER+/HER2− breast cancer has been examined in several retrospective cohorts and meta-analyses, most available evidence has been derived from Western populations (22). In contrast, breast cancer in China presents a distinct epidemiological profile with a significantly younger median age (45–55 years) and a higher proportion of premenopausal patients (23, 24). This demographic difference implies a unique hormonal milieu characterized by higher circulating estrogen levels. Given that AR exerts its protective effect by competitively displacing ER from chromatin, the prognostic weight of AR may be magnified in this high-estrogen context. Consequently, our study extends existing evidence by validating AR’s applicability not just geographically, but within a distinct biological context of a younger, real-world Chinese population.

In the neoadjuvant setting, our observations align with the concept of the so-called “luminal paradox, “ in which lower sensitivity to chemotherapy does not necessarily translate into inferior survival outcomes in luminal breast cancer. In the present study, patients with higher AR expression tended to exhibit lower chemosensitivity, yet showed more favorable disease-free survival, although this observation should be interpreted cautiously given the limited sample size. This phenomenon has been previously described by Loibl et al. in the GeparTrio trial, where AR-positive tumors were less likely to achieve pCR, while AR positivity remained associated with improved DFS and OS (15). These findings collectively suggest that the prognostic significance of AR expression in luminal breast cancer may not be adequately captured by pCR alone. Unlike highly proliferative subtypes such as triple-negative breast cancer, luminal-type tumors often display a more indolent biological behavior. Resistance to chemotherapy in this context may reflect lower proliferative activity rather than aggressive tumor biology. High AR expression has been associated with a more differentiated phenotype and lower Ki67 levels, which may partly explain the reduced chemosensitivity yet relatively favorable clinical course observed in AR-high tumors. Accordingly, the lack of pCR in AR-high tumors should not be interpreted as an unequivocal indicator of poor prognosis. Our findings in the non-pCR subgroup should therefore be regarded as exploratory and hypothesis-generating, suggesting that AR expression may have potential value in refining risk stratification among patients with residual disease after neoadjuvant chemotherapy, rather than serving as a basis for definitive treatment de-escalation decisions.

Our exploratory subtype-stratified analyses further suggest that the prognostic relevance of AR is not uniform across luminal breast cancer. In the present cohort, the favorable association between higher AR expression and survival was more apparent for DFS in Luminal B tumors, whereas no clear prognostic separation was observed in Luminal A tumors. This pattern is biologically plausible, as Luminal B breast cancers generally show lower hormone receptor expression, higher proliferative activity, and a greater risk of recurrence than Luminal A disease, potentially making the balance between AR-related and estrogen-driven signaling more clinically relevant in this subtype. In this context, our findings are partly consistent with the study by Yang et al., who analyzed 985 patients with Luminal B (HER2-negative) breast cancer and reported that high AR expression was associated with better DFS and OS, whereas low AR expression below 65% identified a poorer-prognosis subgroup (25). Although the AR cutoff used in that study differed from ours, both datasets support the notion that AR may have greater prognostic relevance in biologically more aggressive luminal disease. In our cohort, the absence of a significant OS difference after subtype stratification likely reflects the short follow-up duration and the very limited number of death events, particularly in Luminal A tumors. Therefore, these subtype-specific findings should be interpreted as exploratory and require further validation in larger cohorts with longer follow-up.

In the overall cohort, AR was significantly correlated with DFS, whereas no statistically significant difference in OS was observed within the current follow-up period, despite a visible separation of the survival curves. Such differences are not uncommon in the study of LBC, as this subtype has a longer natural course and salvage endocrine therapy is usually effective after recurrence. This finding is not unexpected in luminal breast cancer, which is characterized by a prolonged natural history and a propensity for late recurrence, often occurring beyond the first decade after diagnosis. In addition, effective salvage endocrine therapies after recurrence may further attenuate early differences in OS, particularly in patients with hormone receptor–positive disease.

As Ricciardelli et al. pointed out, the prognostic impact of androgen receptor expression in luminal breast cancer often requires extended follow-up—frequently exceeding 10 years—to translate into statistically significant differences in OS (26). Consistent with this observation, the relatively short follow-up duration and the low number of death events in our cohort likely limited the statistical power to detect OS differences. Therefore, the present study was not designed to draw definitive conclusions regarding the association between AR expression and OS, and all survival findings should be interpreted as reflecting short- to intermediate-term outcomes. Longer follow-up of this cohort will be essential to determine whether the observed DFS advantage associated with higher AR expression translates into long-term survival benefit.

Meanwhile, several limitations of this study should be acknowledged. First, this was a retrospective, single-center study, which may have introduced inherent selection bias and limited the generalizability of the findings. Second, there is no consensus regarding the optimal cutoff for AR positivity in breast cancer, with reported thresholds ranging from low-percentage cutoffs to semi-quantitative H-score–based approaches (21). Although a 1% cutoff is commonly adopted by analogy to ER and PR assessment guidelines, mechanistic studies have demonstrated that AR suppresses estrogen receptor signaling by competitively displacing ER from chromatin binding sites, a process that likely requires sufficiently high AR expression levels (18, 27). Based on this biological rationale, a predefined cutoff of 30% was applied to identify tumors with biologically meaningful AR activity rather than focal or weak expression.

Sensitivity analyses treating AR expression as a continuous variable showed a directionally consistent but non-significant association with DFS, which may reflect limited statistical power due to the small number of recurrence events and suggests that the prognostic effect of AR may not follow a strictly linear dose–response relationship. Importantly, this analysis mitigates concerns regarding *post hoc* cutoff selection and supports the robustness of the observed association. In addition, the follow-up duration of the present cohort was relatively short, and the number of death events was very limited. Consequently, the study was not sufficiently powered to permit a robust assessment of overall survival or breast cancer-specific survival, and the effect of AR expression on long-term prognosis should therefore be interpreted with caution. Further validation in larger cohorts with longer follow-up and more mature survival data is warranted.

In conclusion, this study provides real-world, population-specific evidence from a Chinese cohort supporting the prognostic relevance of androgen receptor expression in ER+/HER2− breast cancer, particularly with respect to disease-free survival within the current follow-up period. Our findings suggest that AR expression may contribute to risk stratification in selected clinical contexts, including patients with residual disease after neoadjuvant chemotherapy, although these observations should be interpreted cautiously. Prospective, multicenter studies with longer follow-up are warranted to further clarify the clinical utility of AR assessment before its routine implementation in clinical practice.

## 5 Conclusion

This single-center, real-world study provides population-specific evidence from a Chinese cohort supporting the prognostic relevance of AR expression in ER+/HER2− luminal breast cancer. Higher AR expression was associated with improved disease-free survival within the current follow-up period, whereas no definitive association with overall survival was observed. In the neoadjuvant setting, exploratory analyses suggested that AR expression may be associated with disease-free survival among patients with residual disease after chemotherapy, although these findings should be interpreted cautiously given the limited sample size and retrospective design. Taken together, our results suggest that AR expression may contribute to prognostic risk stratification in selected clinical contexts, but prospective, multicenter studies with longer follow-up are required to further clarify its clinical utility before routine implementation in treatment decision-making.

## Data Availability

The datasets presented in this article are not readily available because this study is based on confidential patient records collected retrospectively at our institution. Due to strict privacy protections, ethical restrictions, and institutional policies, the raw individual-level data cannot be shared with third parties. Summary data relevant to the results are presented within the article. Further details may be available from the corresponding author, contingent upon ethical approval and compliance with data protection regulations. Requests to access the datasets should be directed to HY;orsonyu@163.com.
