# Supplementary material for: Prognostic value of androgen receptor expression in ER-positive/HER2-negative breast cancer: evidence from a contemporary Chinese cohort
Source: Front Oncol. 2026 May 11;16:1824532. doi: 10.3389/fonc.2026.1824532 (PMC13199044; doi:10.3389/fonc.2026.1824532)
Supplement: Supplementary file 1 [file DataSheet1.docx]

Supplementary Figure 1. Representative IHC and FISH images. All IHC images were captured at ×200 magnification, and FISH images were captured at ×100 magnification.

1. ER-negative


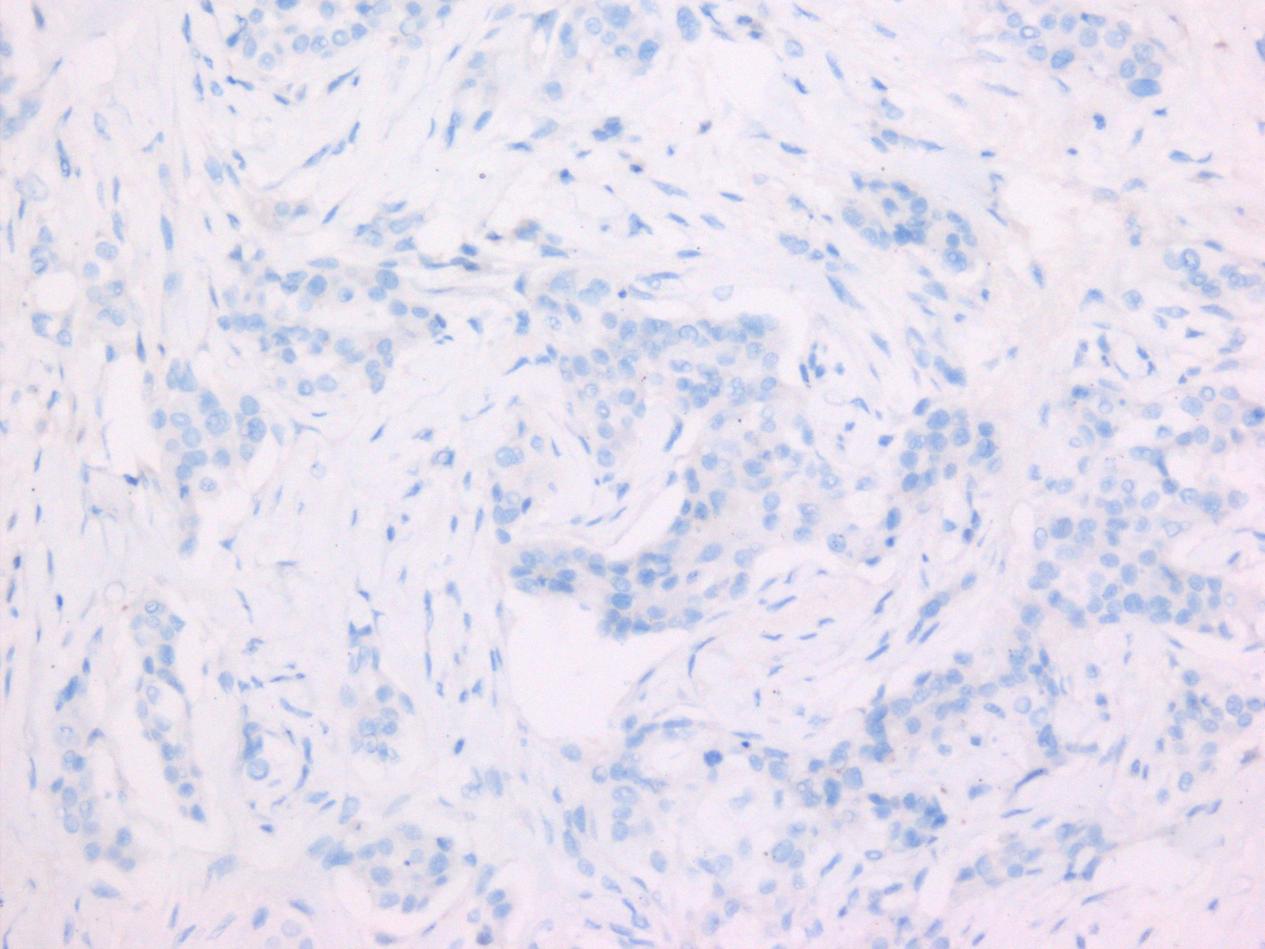


1. ER-positive


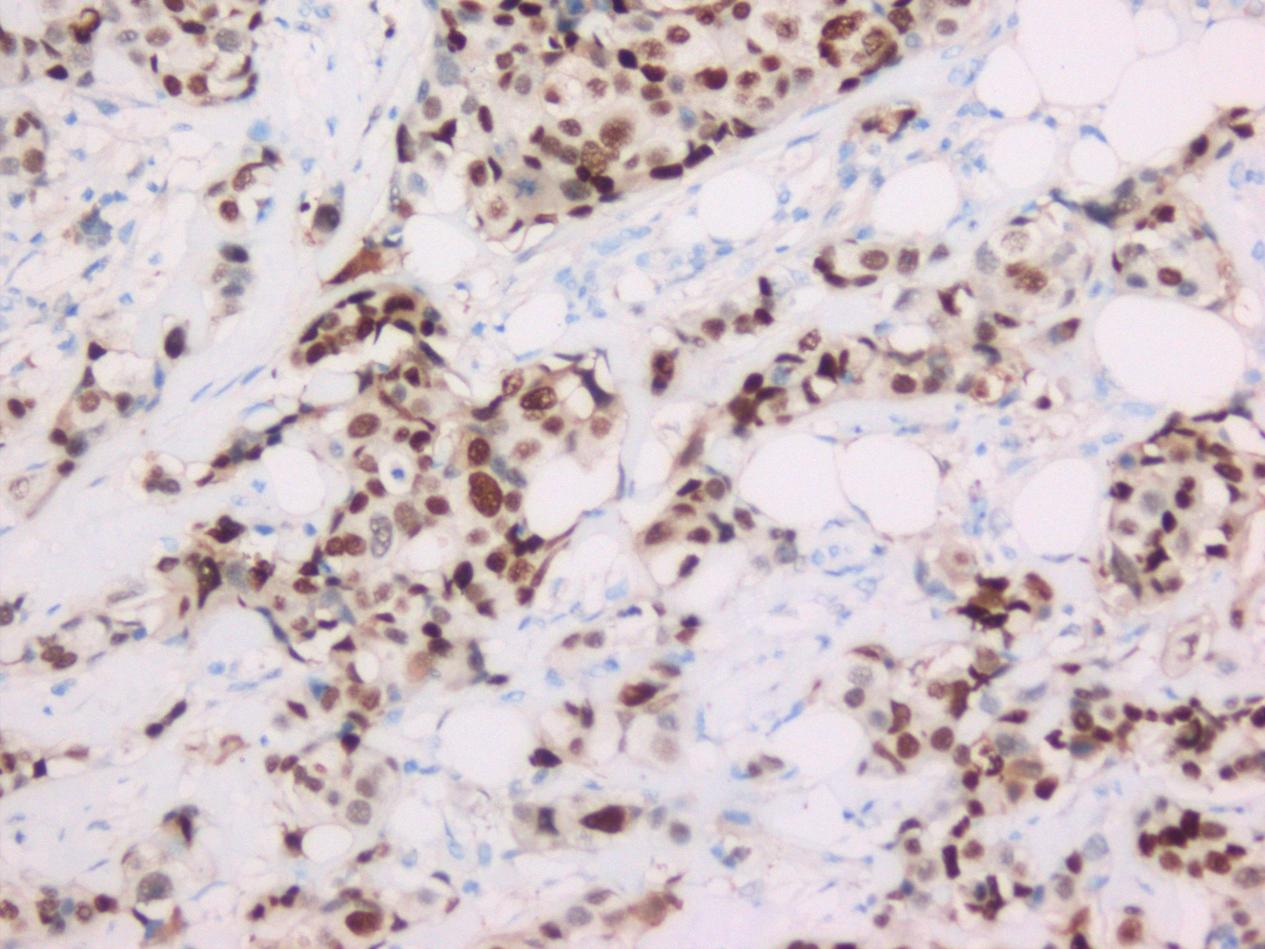


1. PR-negative


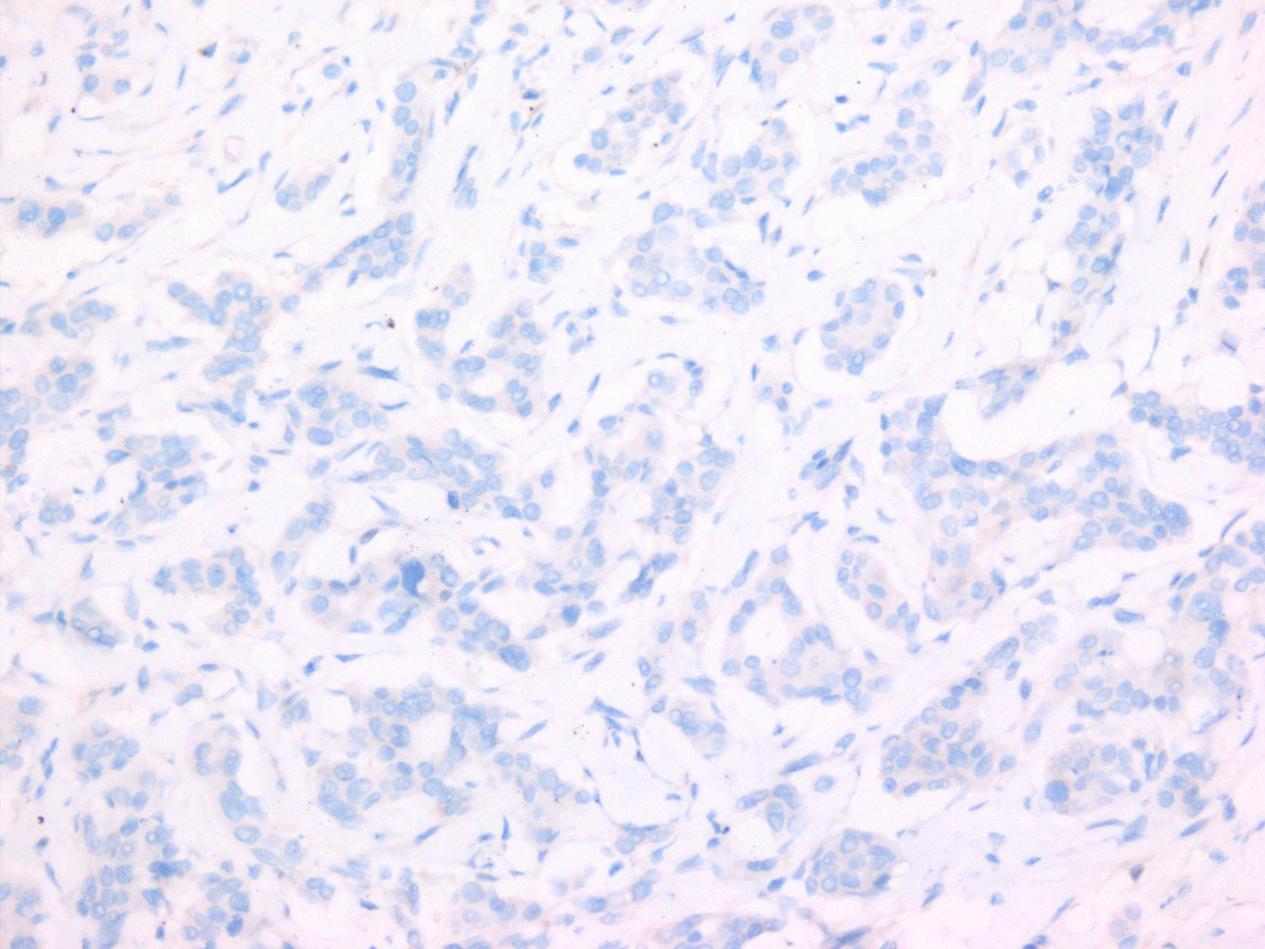


1. PR-positive


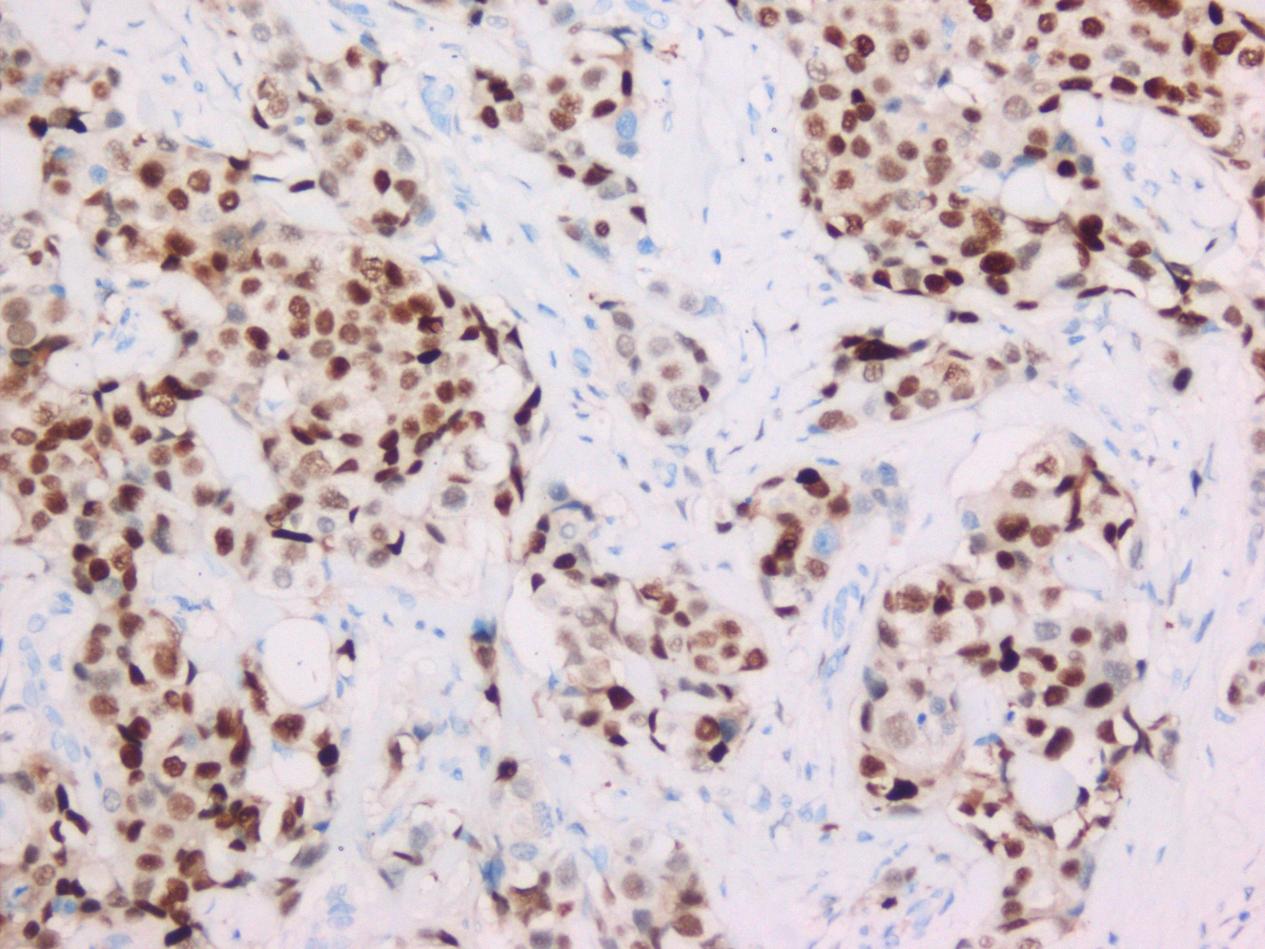


1. HER2 0


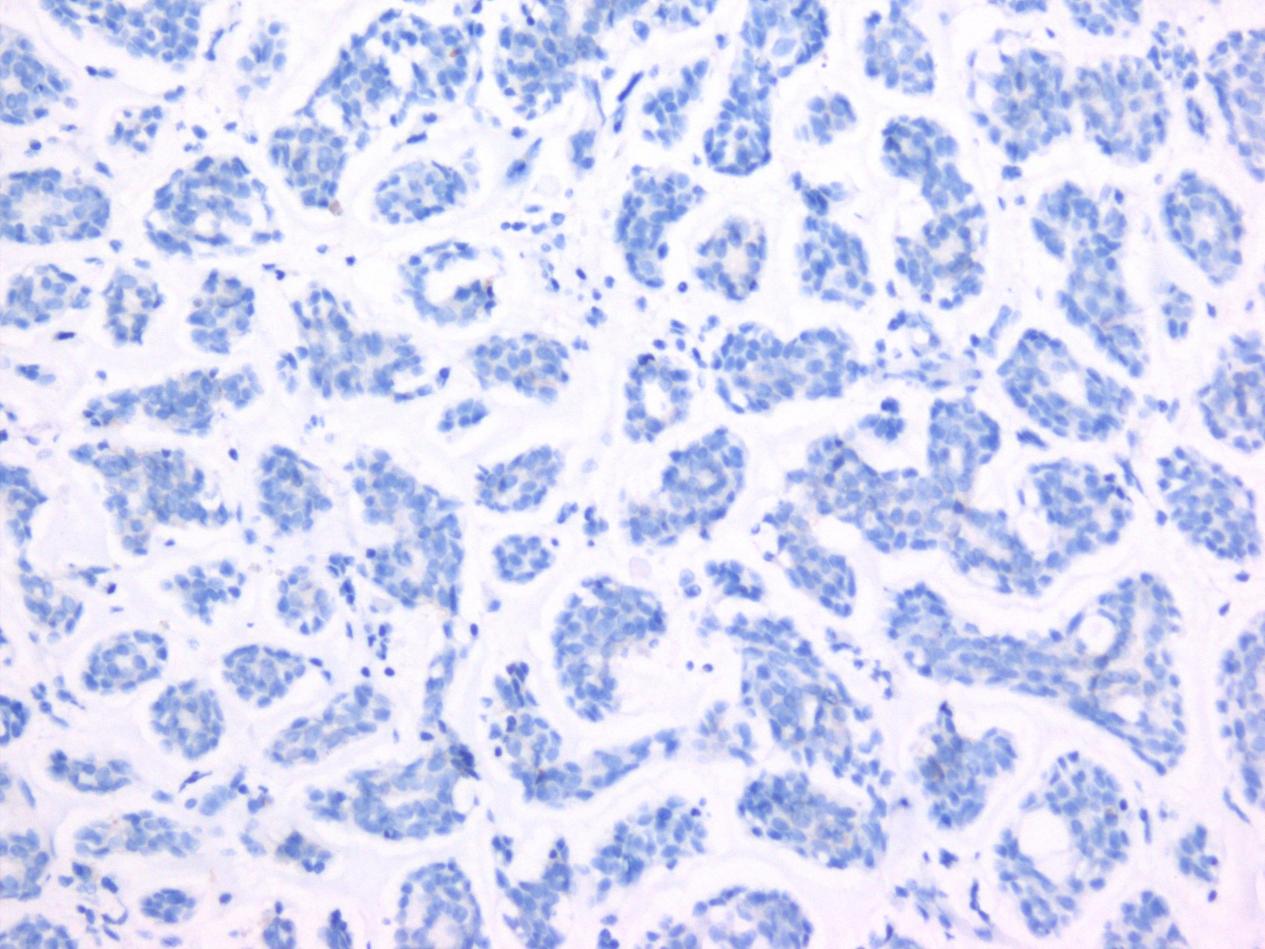


1. HER2 1+


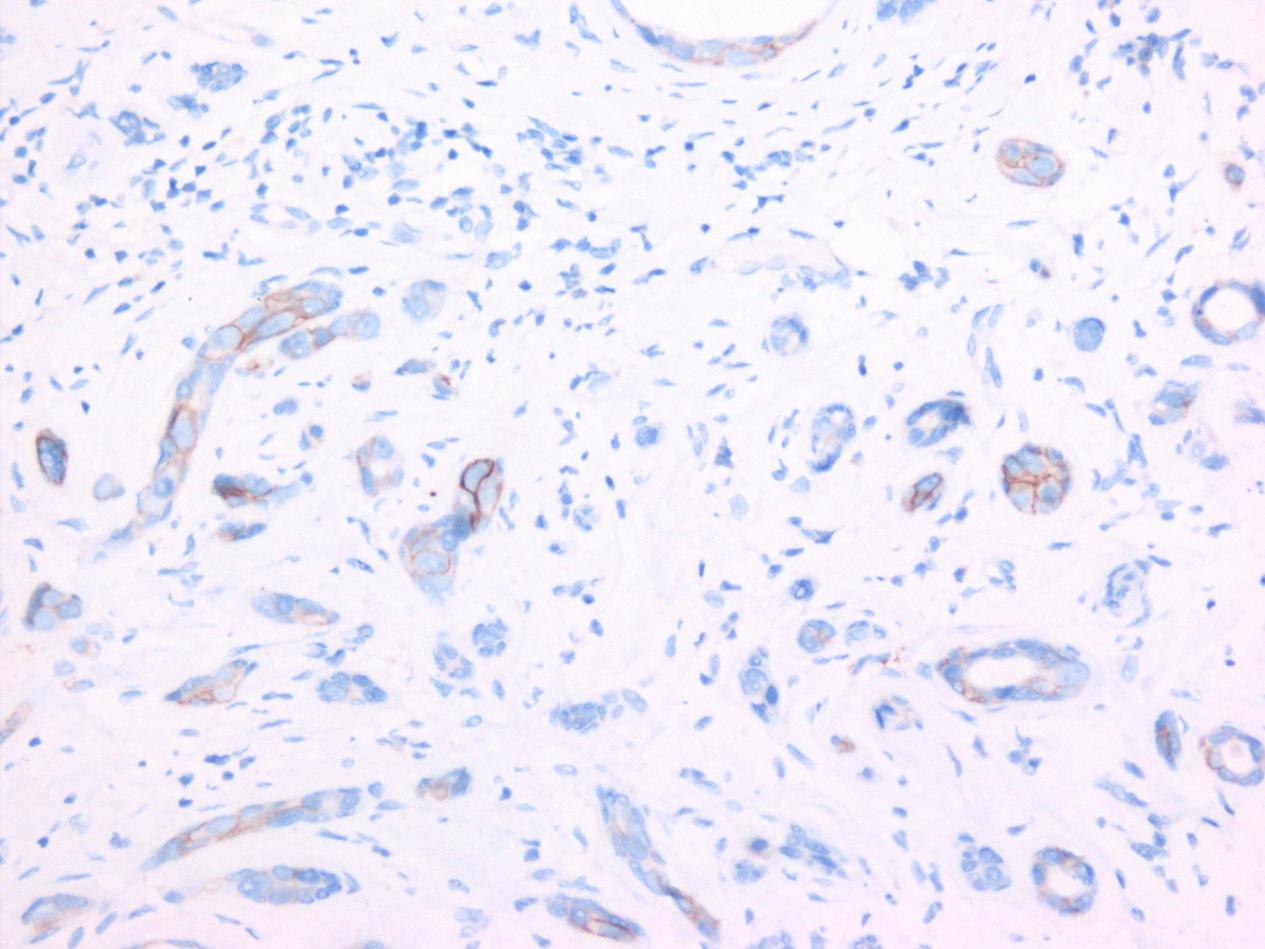


1. HER2 2+


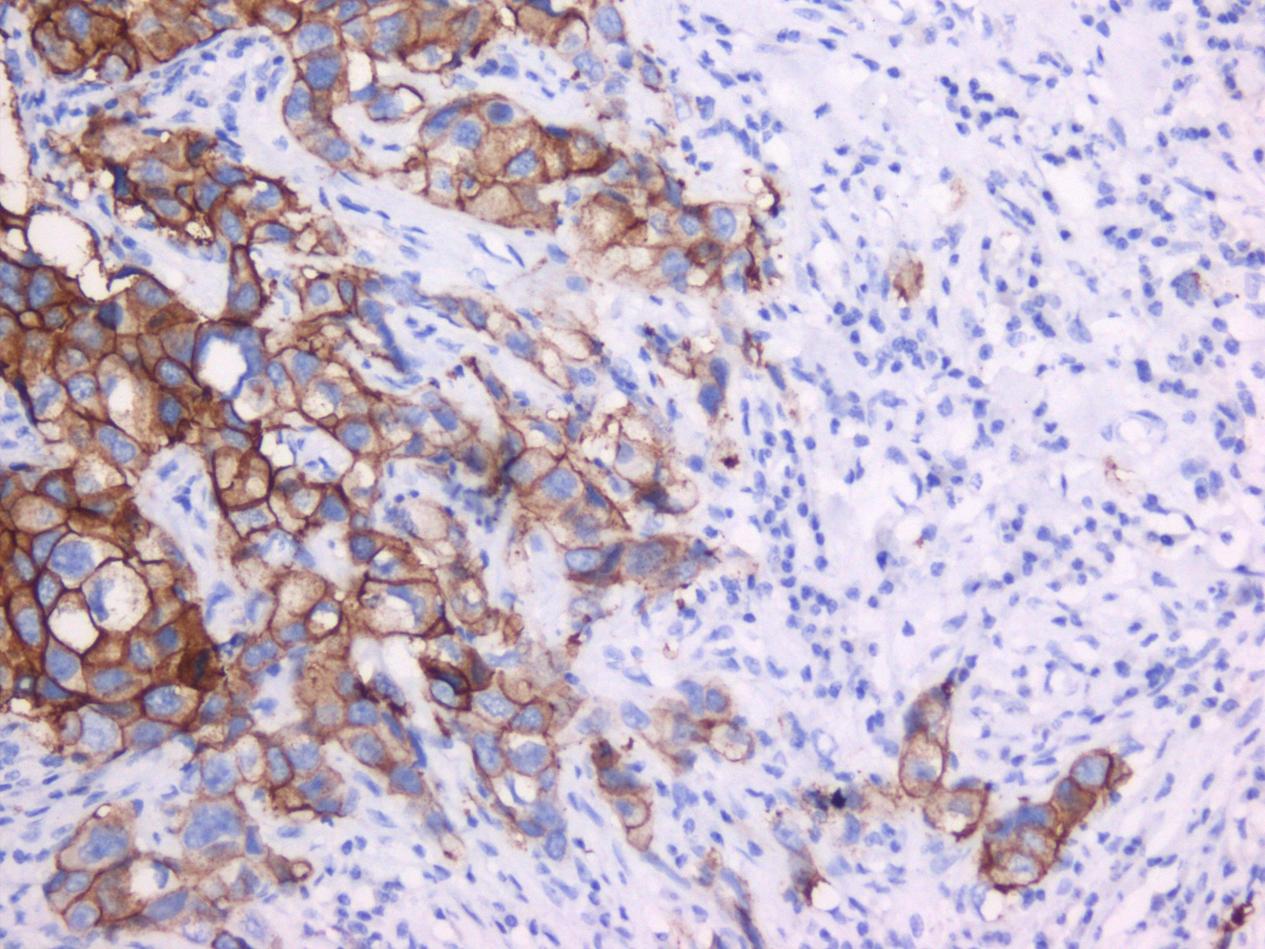


1. FISH-negative result in a HER2 IHC 2+ case


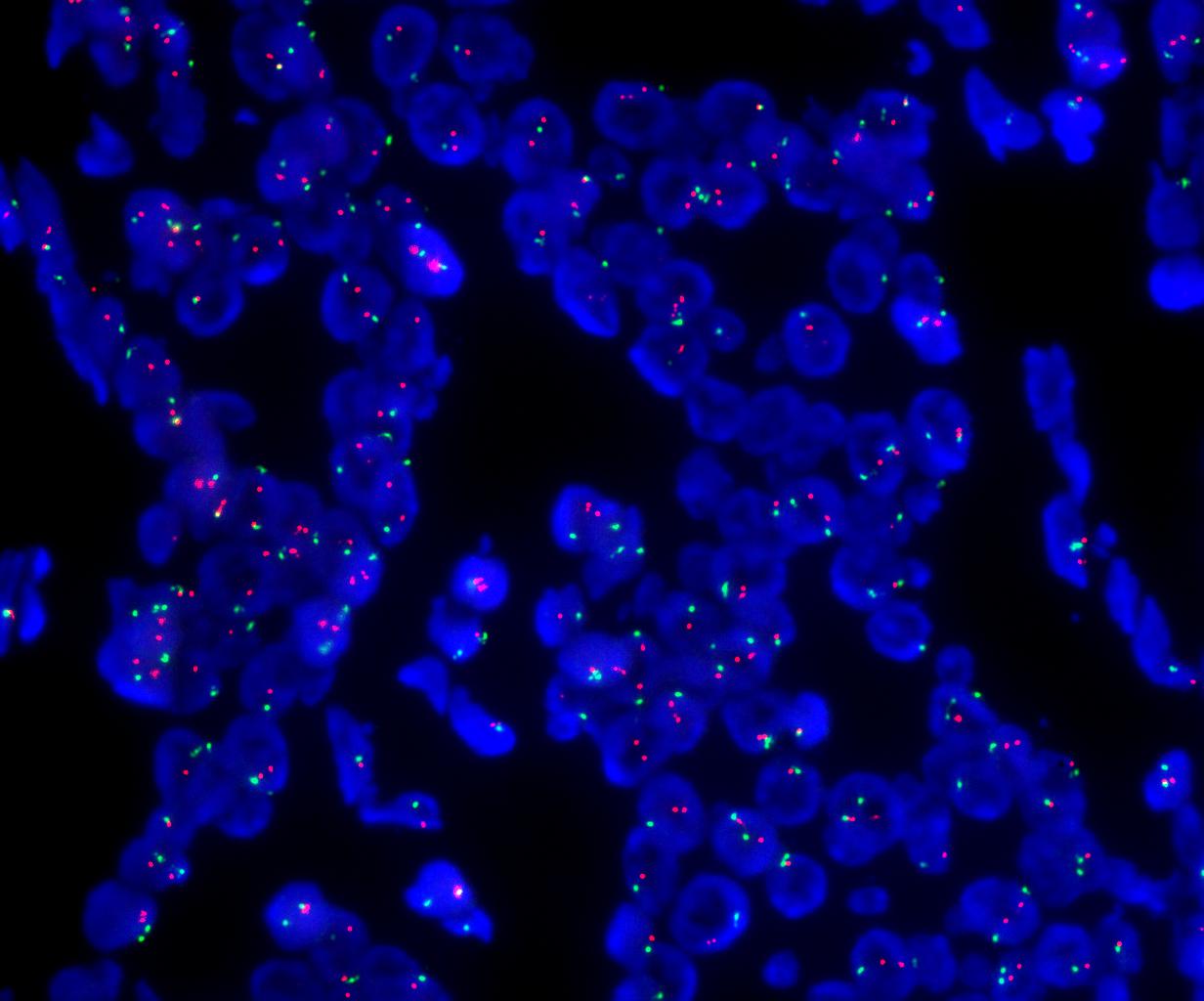


1. FISH-positive result in a HER2 IHC 2+ case


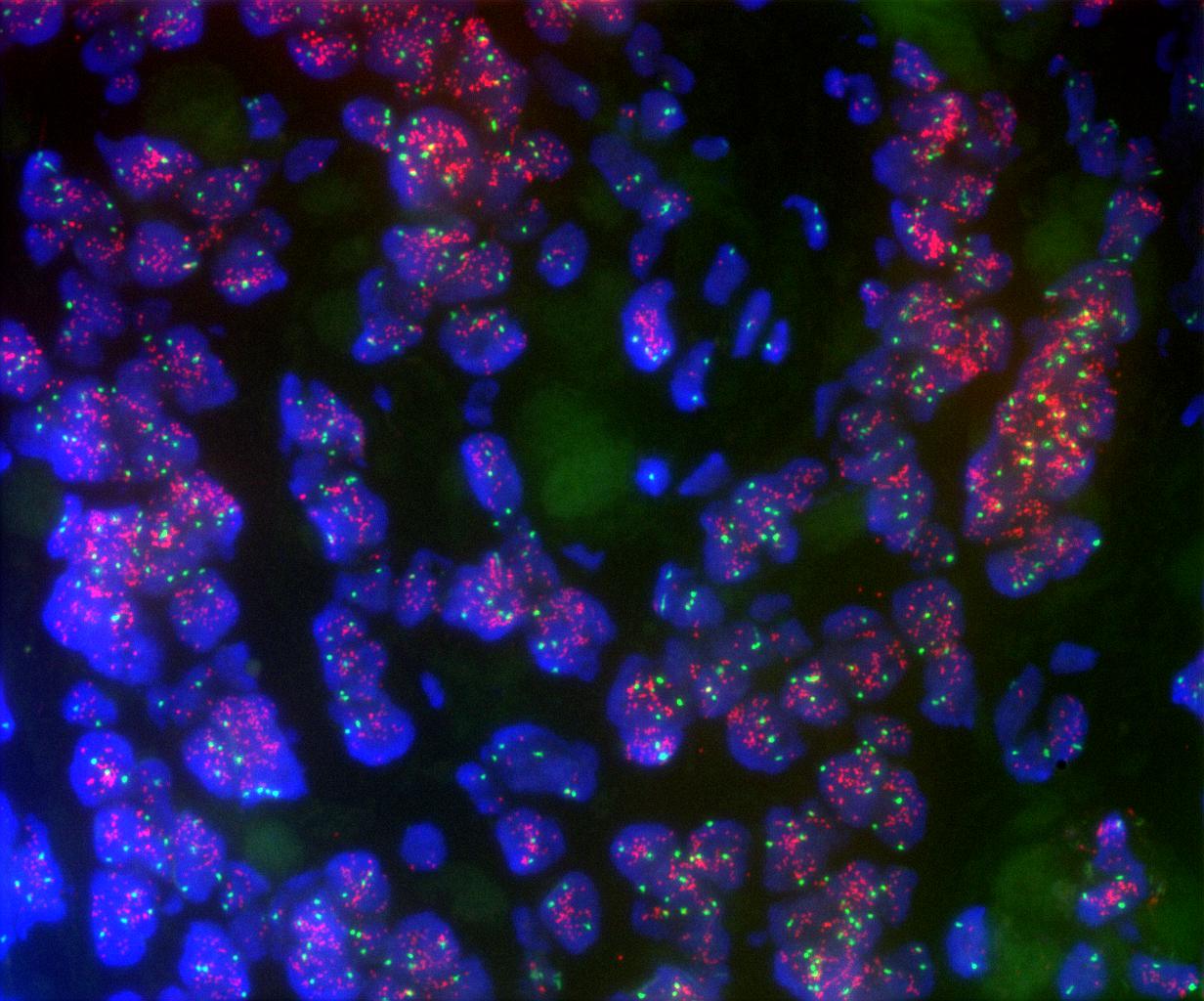


1. Ki-67 >20%


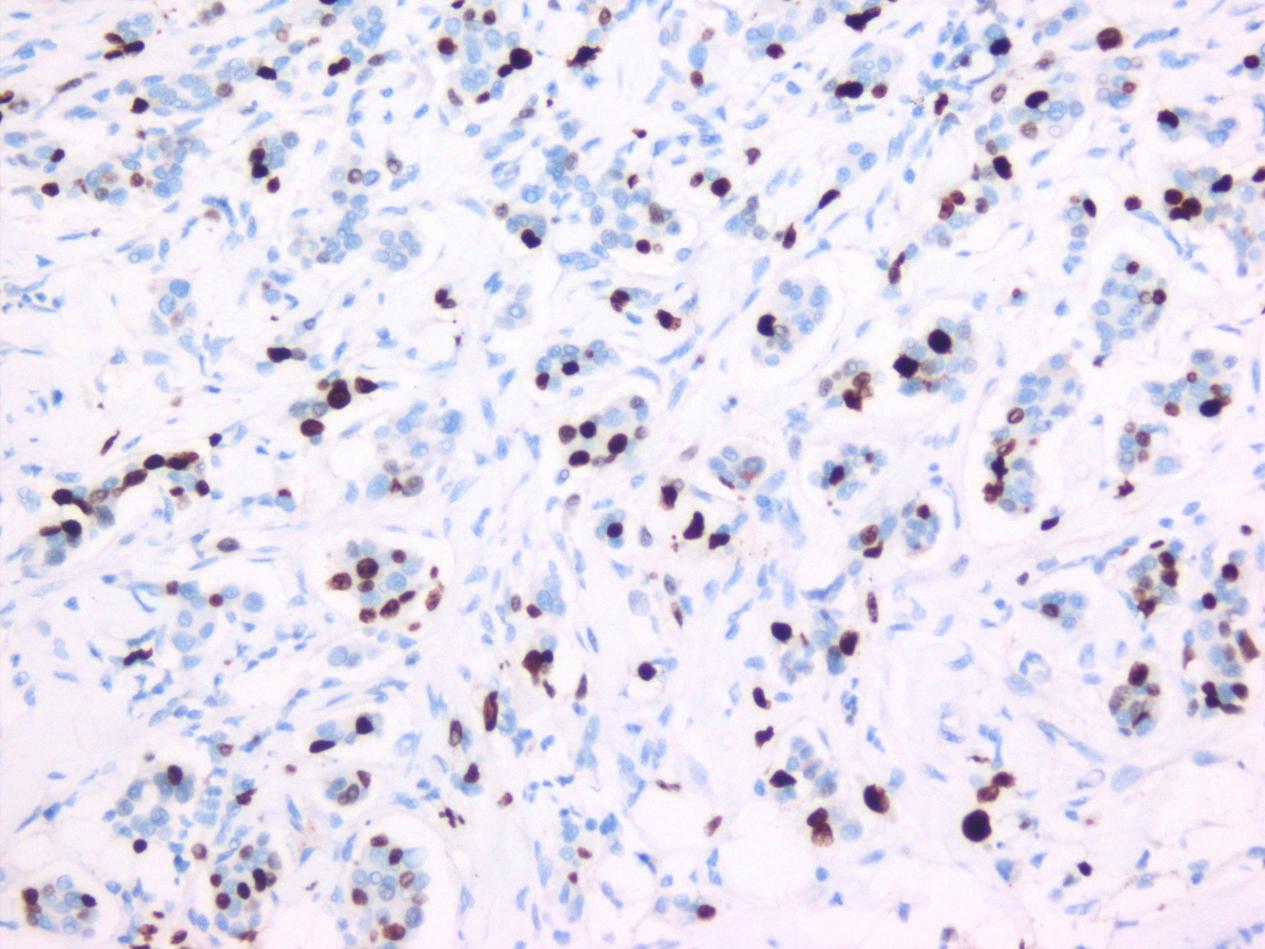


1. Ki-67 ≤20%


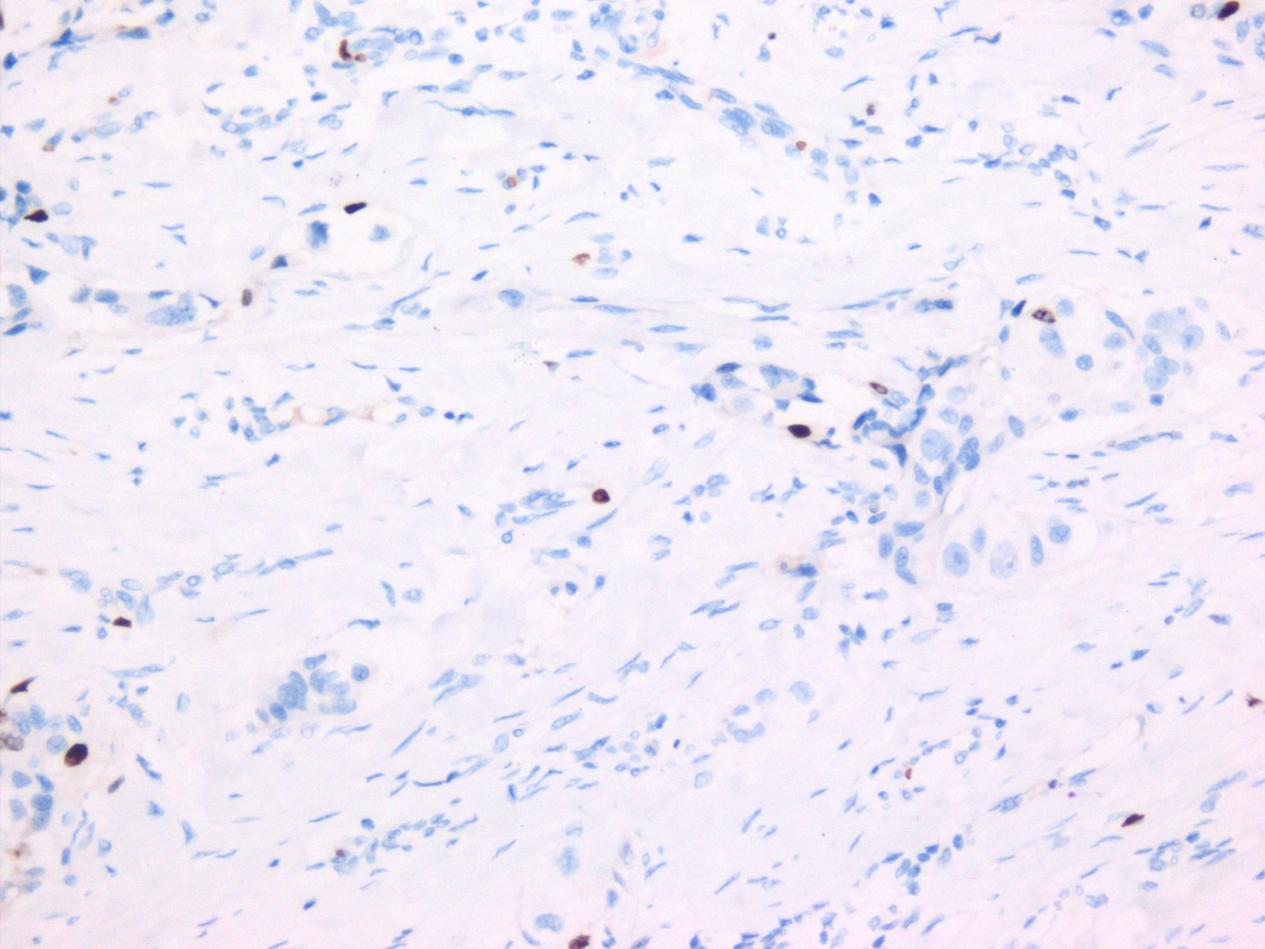


1. AR-positive


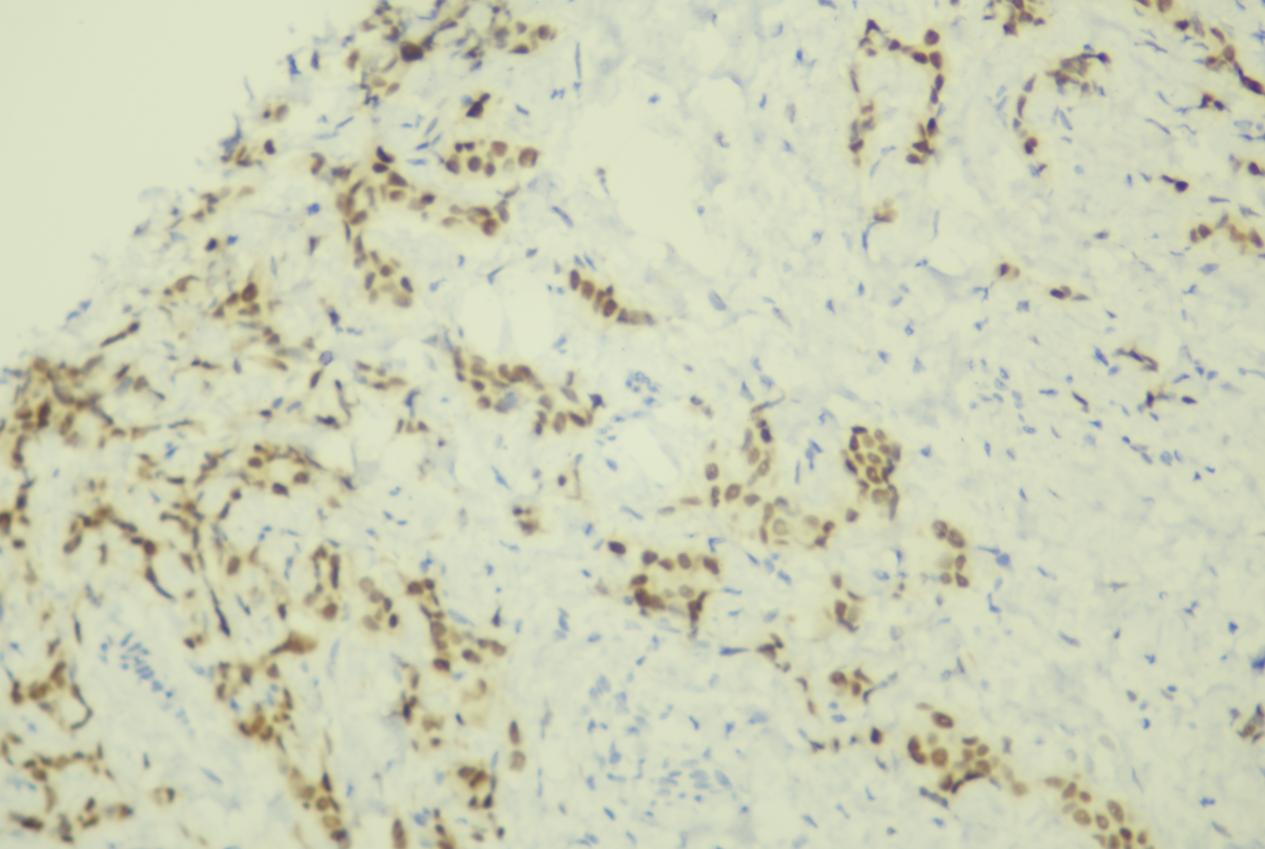


1. AR-negative


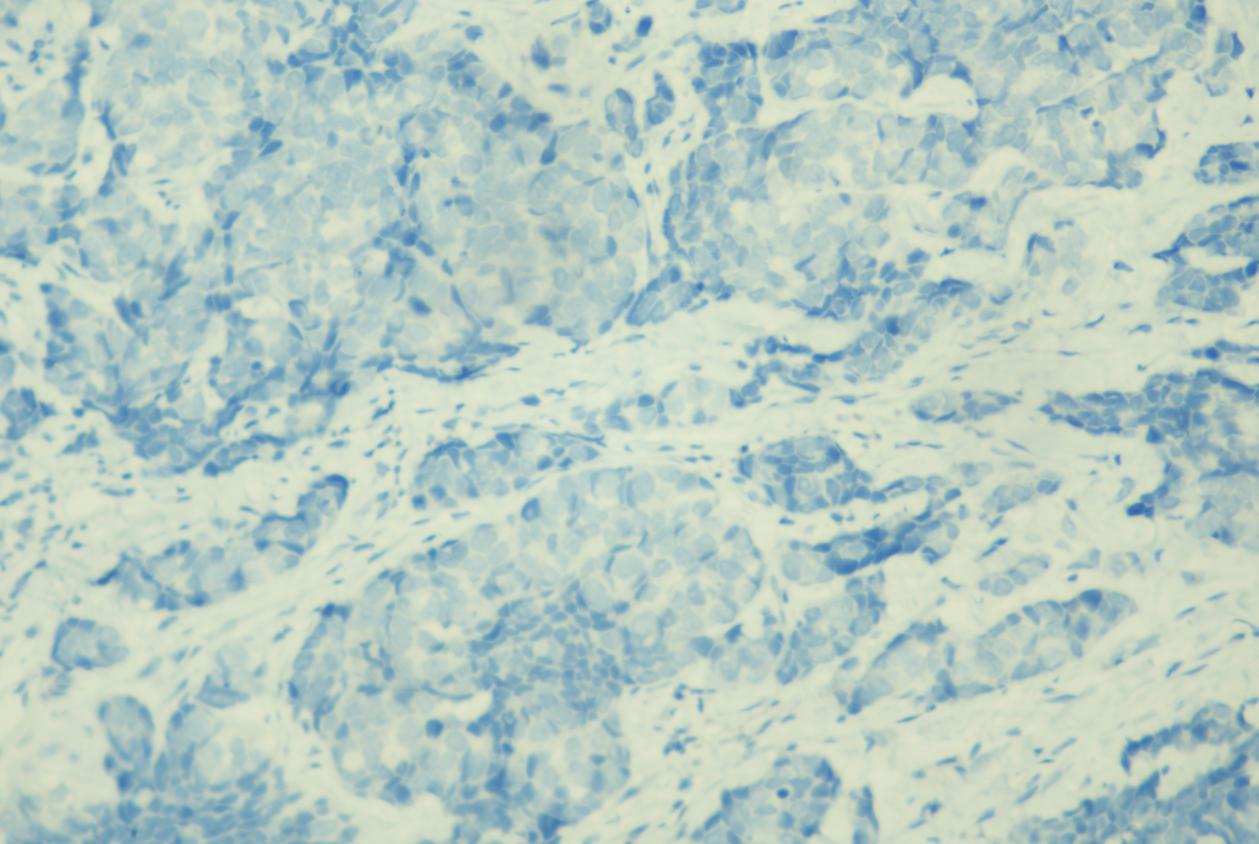


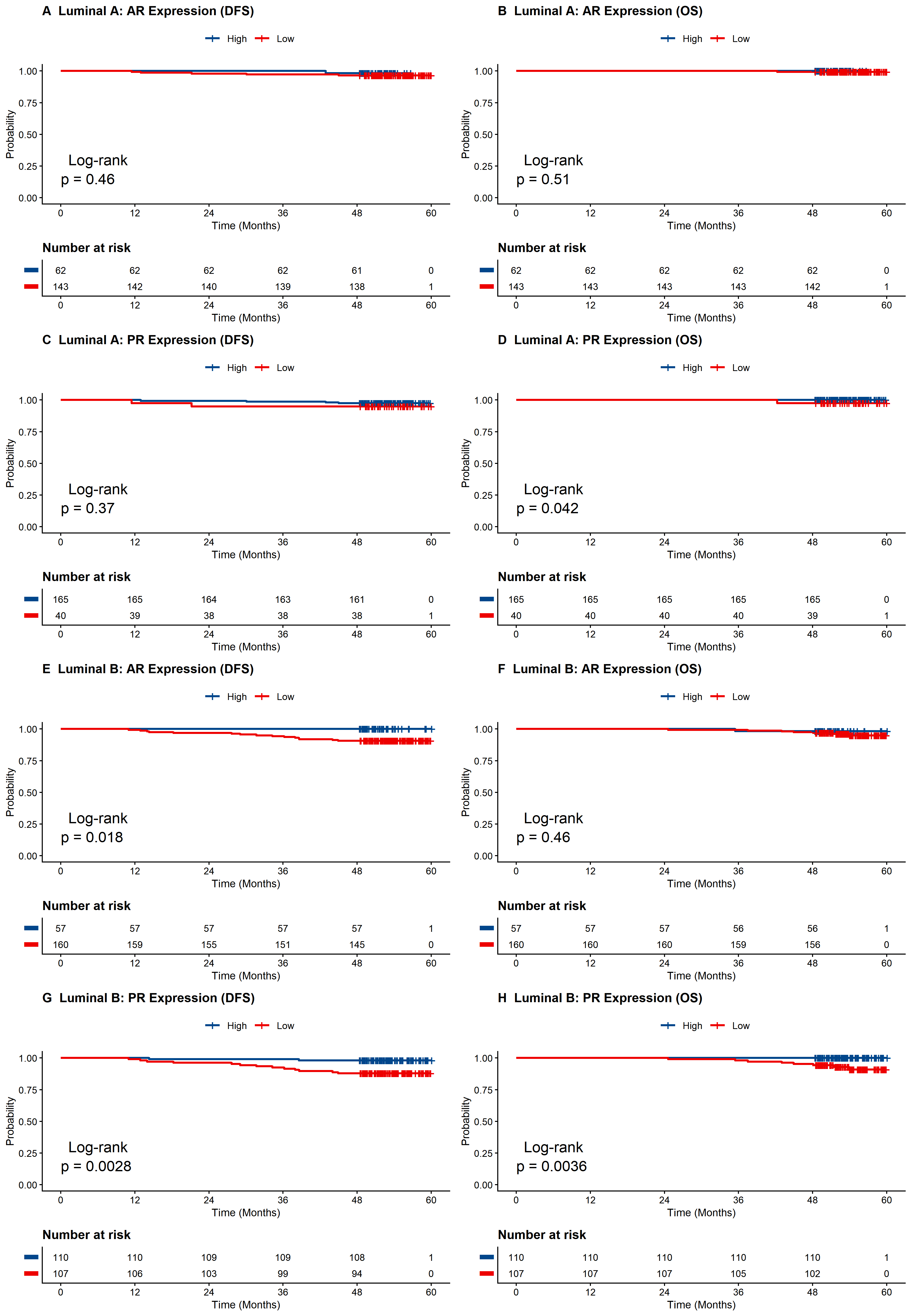


Supplementary Figure 2. Subtype-stratified Kaplan-Meier analyses of disease-free survival (DFS) and overall survival (OS) according to AR and PR expression in luminal breast cancer.
(A, B) Kaplan-Meier curves for DFS and OS, respectively, according to AR expression (>30% vs. ≤30%) in Luminal A tumors.
(C, D) Kaplan–Meier curves for DFS and OS, respectively, according to PR expression (>30% vs. ≤30%) in Luminal A tumors.
(E, F) Kaplan-Meier curves for DFS and OS, respectively, according to AR expression (>30% vs. ≤30%) in Luminal B tumors.
(G, H) Kaplan-Meier curves for DFS and OS, respectively, according to PR expression (>30% vs. ≤30%) in Luminal B tumors.
AR, androgen receptor; PR, progesterone receptor; DFS, disease-free survival; OS, overall survival.
